# A key feedback loop: building electricity infrastructure and electrifying metals production

**DOI:** 10.1098/rsta.2023.0234

**Published:** 2024-11-04

**Authors:** Katrin E. Daehn, Antoine Allanore, Elsa A. Olivetti

**Affiliations:** ^1^ Materials Research Laboratory, Massachusetts Institute of Technology, Cambridge, MA, USA; ^2^ Department of Materials Science and Engineering, Massachusetts Institute of Technology, Cambridge, MA, USA

**Keywords:** energy transition, extraction, electrolysis, energy consumption, processing, electrification

## Abstract

Energy infrastructure requires metals, and metals production requires energy. A transparent, physical model of the metals-energy system is presented to explore under what conditions this dependence constrains or accelerates the transition to a net-zero economy. While the mineral (as high as 340 Mt yr^−1^ iron ore, 210 Mt yr^−1^ limestone, 250 Mt yr^−1^ bauxite and 5.5 Gt yr^−1^ copper ore in the 2040–2050 decade, assuming no improvements) and total energy (up to 22 EJ yr^−1^) requirements for building low-carbon energy infrastructure are significant, it compares favourably with the current extraction and energy use supporting the fossil fuel system (15 Gt yr^−1^ fossil minerals and ~38 EJ yr^−1^). There are levers to significantly reduce material use and associated impacts over time. The metals industry can play a key reinforcing role in the transition by adapting to the increasing supply of renewable electricity. Specifically, direct electrolysis can extract metal from ore close to the thermodynamic limit, to make efficient use of low-C electricity. The unique features of emerging technologies for iron extraction, molten oxide electrolysis and molten sulphide electrolysis are considered in this evolving system. Electrification enables elegant separations and provides a pathway to build out infrastructure while reducing environmental impacts, though material efficiency measures will still be crucial to meet 2050 carbon budgets.

This article is part of the discussion meeting issue ‘Sustainable metals: science and systems’.

## 1. Introduction

As the physical economy disentangles from fossil fuels and scales up low- or zero-carbon resources, new connections and relationships will emerge between sectors. The IPCC calls for policies and models to consider such interconnections, synergies and trade-offs among and within sectors, which is not currently the norm [1]. The coupling between energy and metal production is inherent and central to the transition to a low-carbon economy. Metals are required to generate, store and transmit energy. The production of metal from ore is energy intensive. Disruptions in the transition to a zero-carbon economy may emerge if one industry cannot meet the demands of the other, while simultaneously aggressively cutting carbon emissions. When could this coupling result in carbon-intensive lock-in, or is there potential to transform both industries together?

### (a) Material constraints for building energy infrastructure

Literature addresses the materials required to build a net-zero energy system, driven by concerns over the vast infrastructure required to generate and transmit electricity from wind and solar sources. Existing literature quantifies the bulk and critical materials required per generation capacity, or unit of electricity generated, for a range of technologies. Watari *et al*. [2], Deetman *et al*. [3], Elshkaki *et al*. [4], Manberger *et al*. [5], Wang *et al*. [6] and Kalt *et al*. [7] have performed recent dynamic scenario analyses for the materials requirements to build a net-zero or low-carbon energy system. While there is significant uncertainty, the emerging consensus is that there are no firm material constraints. Indeed, improvements in efficiency and material use can significantly reduce requirements over time [8,9]. Scaling up the supply of a selection of critical metals in a timely, socially and environmentally responsible manner remains a key challenge [10]. Still, commodities such as steel, concrete, aluminium and copper will account for the vast majority of the CO_2_ impacts [6].

While total material requirements are unlikely to limit the build-out of low-carbon energy infrastructure, the CO_2_ intensity and energy intensity of their production can still undercut the benefits and feasibility of such a transition [11]. To extract metal from ore, energy is required, which may be provided in the form of fossil fuel or electricity. Steel dominates the metals industry (nearly 2Gt yr^−1^ produced globally), with aluminium (approx. 100 Mt yr^−1^) and copper (approx. 23 Mt yr^−1^) produced at smaller scale, but still categorized as commodity or ‘bulk’ metals. Steelmaking in the blast furnace uses coke and pulverized coal as a reducing agent, providing chemical work and heat, and simultaneously carbon as an alloying element to make steel. CO_2_ (1.7–2 t/t-steel) is generated as oxygen, from the ore and some blown additionally, reacts with the carbon in coke and coal. To make aluminium, bauxite ore is pre-treated in the Bayer process to remove gangue (e.g. silica, hematite and goethite) and other impurities, which is typically powered by coal (approx. 12 GJ t-Al^−1^ [12]) and leaves highly alkaline tailings (2–3 t t-Al^−1^ [13]). The alumina is then dissolved in a molten cryolite electrolyte, and electricity is used to decompose it into molten aluminium at the cathode and CO_2_ (1.5–2 t CO_2_-eq t-Al^−1^) at the carbon anode. Indirect emissions from electricity contribute most significantly: the global average is 7−10 t CO_2_ t-Al^−1^, but up to 15 t CO_2_ t-Al^−1^ results from coal-fired electricity [14]. Copper is produced primarily via oxygen smelting of chalcopyrite. SO_2_ is the main by-product, but CO_2_ results from electricity and fuel use during smelting and in the many upstream and downstream ancillary processes. Copper ore grades are expected to decrease to 0.2–0.4% Cu on average by 2050, which will increase the energy demand for mining and beneficiation significantly [15]. Fizaine & Court [16] present a scenario where the energy required to extract metals (copper, nickel, gold, etc.) increases and warns against a vicious circle developing between energy and metal sectors, where metal production requires more energy, further increasing the demand for energy infrastructure and metals. By contrast, Harpprecht *et al*. [17] modelled the supply of metals to 2050 dynamically and found significant opportunities to reduce environmental impact over time.

### (b) Energy constraints for building energy infrastructure

The energy intensity of the materials behind solar panels, wind turbines and the grid has led to concern about whether too much energy will be indirectly diverted back to the energy industry, with a reduced surplus for the rest of society. Every energy source (e.g. coal-fired power plants and oil, bioenergy) requires an energy investment to build and maintain. The metric ‘energy returned on energy invested’ (EROI) has been defined as the ratio of useful net energy yielded from each unit of energy input to the process of obtaining that energy. It has been used to compare different energy sources and evaluate the feasibility of transitioning to solar and wind [18]. King *et al*. [19] factored in a range of EROI estimates of different energy sources in the International Energy Agency (IEA) renewable energy scenarios and found a reduction in net energy to society, significant enough to threaten current lifestyles. Capellan-Perez *et al*. [20] proposed an integrated assessment model with biophysical constraints and found a large penetration of renewables drives a large requirement for minerals, energy investments and land such that a persistent recession will be caused by energy scarcity and climate damages. Vidal *et al*. [21] warn against a vicious circle forming when replacing fossil fuels with metals and minerals.

The EROI metric can be easily manipulated, depending on the assumptions and system boundaries [22]. The differing thermodynamic qualities and quotidian practicalities between the forms of energy—electricity, heat and fuel—are difficult to capture in a single metric. Electricity offers much higher conversions to useful energy, but fuels have the advantage of being available in discreet, dispatchable quantities, to be burned when needed. Nevertheless, an understanding of the order of magnitude of the total energy required to build out low-carbon energy infrastructure and how that compares with the energy used to operate the system today would be helpful to indicate feasibility and is not available in the literature.

### (c) Low-carbon electricity constraints for metals production

The whole economy is under the constraint to meet a carbon budget to stay well below 2°C warming. The metals industry is expected to meet increasing demand while cutting emissions [23]. Watari *et al*. [24] envisioned a transition in steelmaking towards scrap-based and hydrogen-based direct reduction routes. Future access to carbon capture infrastructure and low-carbon electricity was constrained within a set window, informed by historic growth rates and IEA projections. With these limitations, it was found only 58–65% of the baseline global steel demand could be met. Delivering the same service with much less steel is possible and requires urgent attention. The metals industry’s licence to operate in a carbon-constrained world depends on access to low-carbon electricity, as well as the development of technologies to utilize this resource efficiently.

Clearly, the fates of the energy and metals industries are tied. While many roadmaps for a net-zero energy system have been proposed, an equally bold transformation for the metals industry is not as widely anticipated. Steelmaking is categorized as ‘hard-to-abate,’ with end-of-pipe carbon capture and sequestration as the key intervention for decarbonization. However, the energy transition will increase the supply of low-carbon electricity, a powerful vector for materials processing—providing a finite and controllable difference in chemical potential. Birat [25] explains that there are many technological opportunities to utilize more electricity in materials production, but the high price of electricity has thus far curbed its use. The direct electrification of steelmaking, in a configuration similar to the Hall–Héroult cell, has been demonstrated and is nearing commercial implementation. The Hall–Héroult process enabled a rapid reduction in specific energy consumption and an increase in cell productivity in the last century, at rates unseen for any other material process [26]. While the energy consumption of blast furnace–basic oxygen furnace (BF-BOF) steelmaking is considered nearly optimized, full electrification may offer a new level of efficiency and processing opportunities.

There are competing trends in the transition to a net-zero economy: the total energy used by the energy industry will likely increase due to infrastructure investments but may be manageable if learning curves continue. Materials production may become less CO_2_-intensive as processes are electrified. Pehl *et al*. [27] and Hertwich *et al*. [28] use an integrated assessment model coupled with life cycle assessment to quantify the material, energy and CO_2_ implications of building low-carbon electricity infrastructure while incorporating incremental efficiency improvements and decreasing emissions intensity. Similarly, Elshkaki [4] explores the global energy transition with a dynamic material flow-stock model that incorporates dynamic intensity factors for energy and CO_2_. Still, these models with updating intensities do not represent the underlying production processes explicitly and structural changes such as the deployment of new process technologies are not anticipated. Here, we present a model of the bulk materials and energy flows associated with building new energy infrastructure and the materials production process routes, to explore the influence of key factors such as ore grade decline, learning curves and the deployment of novel electrified technologies to determine under what conditions constraints arise for the energy and metals industries. When might the material and energy requirements for building new energy infrastructure cause limitations? How powerful is electrification as a lever for decarbonizing the metals industry, and will enough low-carbon electricity likely be available? We quantify the material and energy requirements for the new energy system, as well as the energy requirements and associated emissions for the metals industry across several electrification scenarios. As background to inform the scope and assumptions for future technologies evaluated in the model, the key features of direct electrolysis technologies are first described in §2, before proceeding to methods in §3.

## 2. Features of direct electrolysis

Hydrogen-based steel production and adding carbon capture and sequestration to blast furnaces have received attention as promising decarbonization routes for steelmaking, but both increase energy intensity—to make the hydrogen and provide heat during the endothermic reaction [29], or to scrub, transport and inject CO_2_ [30], respectively—while offering questionable or no process advantages [31,32]. Alternatively, electricity can be used to directly reduce iron ore. As explained by Allanore [26], as early as 1807 chemists saw the opportunity to investigate the ‘true elements of bodies’ using the potential difference of a battery. The development of direct electrification technologies will be modelled in the context of the energy transition towards electricity as the main carrier.

**Figure 1.**
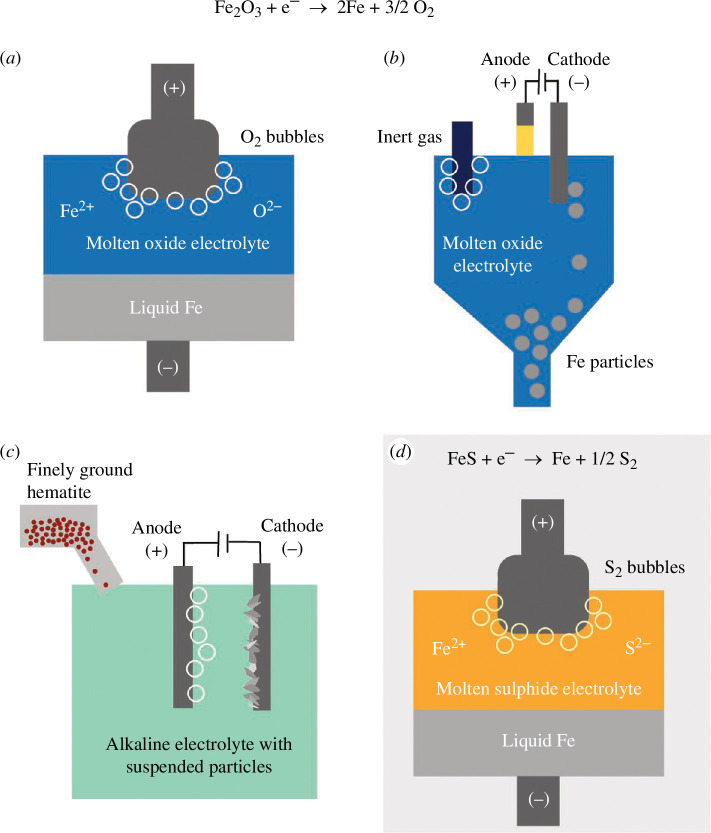
Schematics of electrified iron production technologies: (*a*) molten oxide electrolysis (MOE) with an inert anode, (*b*) MOE with a plasma anode, (*c*) alkaline electrowinning of hematite particles and (*d*) molten sulphide electrolysis (MSE).

To decompose a metal-containing ore to its constituents (the metal cation and the anion, typically oxygen or sulphur) using electricity, a range of set-ups can be imagined. The supporting electrolyte is at the heart: it dissolves the ore (acting as a solvent) and supports ionic conduction of the species such that the cations migrate to the cathode and the anions to the anode under the application of the potential difference [33]. The chemical classes of electrolytes include aqueous, halide, fluoride, oxide and sulphide. The characteristics of these electrolytes considering the challenging benchmarks for commercial commodity extraction are discussed in Allanore [34]. The various configurations summarized here are shown in figure 1.

Generally, low-temperature processes with an aqueous electrolyte exhibit low current densities (and low productivity). However, a unique arrangement has been demonstrated for iron production, where hematite particles are suspended at high concentrations in an alkaline electrolyte to support high current densities (up to 1 A cm^−2^, competitive for commodity production) [35]. Energy consumption has been recorded at 2600 kWh/t_Fe_, close to the thermodynamic minimum. This process is under further investigation and scale up [36].

Operating at high temperature enables liquid metal production and tapping for continuous operation. High current densities can be achieved due to the ability of the molten electrolyte to dissolve the iron ore feedstock at high concentrations (following the principle ‘like dissolves like’). Heat is generated as electrical potential is applied between the electrodes via the Joule effect, to maintain the operating temperature directly and efficiently [37].

Molten oxide electrolysis (MOE) is a high-temperature route where hematite is dissolved in the supporting electrolyte containing silica, alumina, magnesia and calcia [38]. The anode must be electrically conductive and support oxygen evolution while withstanding extremely basic, high-temperature conditions with minimal reaction: ‘the ultimate materials challenge’. The demonstration of an inert anode made of relatively cheap material, Cr–Fe, was a step forward [39] and a pilot scale facility with a cell design similar to Hall–Héroult is underway. An inert anode is not the only possibility: a plasma-based anode [40] or consumable magnetite anode [41] has also been investigated.

We want to highlight one further direct electrolysis pathway: molten sulphide electrolysis (MSE). Sulphide electrolytes have been under-explored, as sulphides can exhibit high electronic conductivity, but Sokhanvran *et al*. [42] found that a supporting electrolyte of BaS was sufficiently ionically conductive to support Faradaic reactions and decompose Cu_2_S to liquid copper and sulphur gas, using a stable graphite anode. This technique has since been demonstrated to decompose chalcopyrite (CuFeS_2_) to produce liquid cast iron and copper in separate steps [43]. This process may help the copper industry adapt to more complex deposits and water stress, as this route uses only sulphur and electricity as levers for unique elemental distributions [44]. Results of cast iron production from pyrite (FeS) with high Faradaic efficiencies have recently been obtained [45].

Extraction of iron in the sulphide regime reduces energy needs by 25% compared with the electrochemical reduction of oxides (a full 60% compared with the blast furnace), while providing novel process opportunities. The Gibbs free energy (∆*G*) to decompose FeS is lower than ∆*G* for Fe_2_O_3_. The valency of iron in the sulphide electrolyte is 2+, also serving to reduce the electricity requirement and side-step the multi-valency inefficiency encountered in iron oxide electrolysis. FeS is abundant, but the reserves have not been quantified because it is considered waste today. The conversion of iron oxide to iron sulphide is also likely practical, as the reaction is exothermic and resulting SO_2_ emissions can be readily captured and scrubbed [46]. As stated above, *cast iron* can be directly produced, which is a key advantage compared with oxide routes (carbon would cause carbo-thermic reduction of the electrolyte in MOE and a solid iron product is formed in aqueous electrolysis). The addition of carbon to iron is crucial not only for steel’s unparalleled strength and versatility but also for melt processing. Carbon lowers the melting point for much easier heat management and reduced refractory wear and is a powerful agent to control the chemistry of the melt; for example, the solubility of oxygen in molten iron decreases as carbon increases. While MSE is under active development and difficult to project into a global market, it is included to draw attention to this under-explored design space.

## 3. Methods

To couple the build-out of low-carbon energy infrastructure with process models of bulk materials production, existing literature on the materials requirements of the energy transition was used. Many scenarios for the energy transition have been explored in the literature, to consider the full range of energy generation technologies [5] and decarbonization timelines. Here, a single energy transition scenario (the 2023 net-zero energy scenario by the IEA) is used, so that the impact of assumptions about the production of the infrastructure can be explored. To set the context, the current energy system is characterized, described in §3a. The baseline case for building the future energy system is defined in §3b, and dynamic changes to this baseline are described in §3c.

### (a) Current energy system

To provide context against which to compare the future energy system, the data from IEA’s 2019 extended world energy balance were compiled and visualized [47]. The major energy inputs were categorized and compared with final consumption, where they enter the economy for use. Details of this summation and values of all flows can be found in the electronic supplementary information.

### (b) Baseline case for building the future energy system

**Figure 2.**
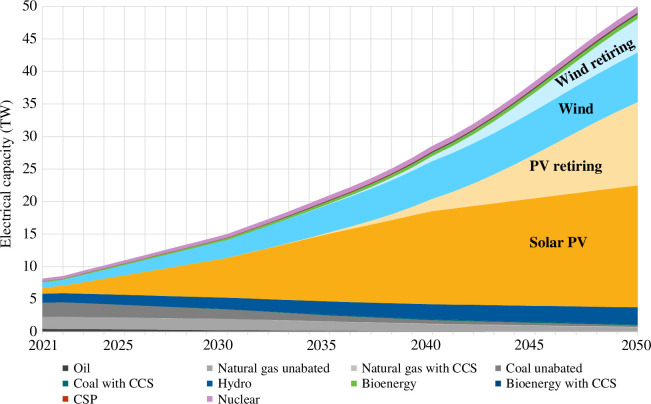
Total electrical capacity (TW) by generation technology from 2021 to 2050, as projected by the IEA NZE [48]. The estimated capacity of PV and wind retiring to 2050, with the lifetime assumptions described in §3c(ii), are plotted as well.

To simulate the build-out of a net-zero energy system to 2050, the IEA’s Net-Zero scenario (2023) [48] was chosen, which has been recently updated to reflect the latest empirical data. The projected global electrical capacity by generation technology from 2021 to 2050 is shown in figure 2. The capacity of wind and solar are assumed to multiply by ~20 and ~9 from the present day to 2050, respectively, to dominate the electricity sector of the future. Modest growth in capacity is anticipated for nuclear, hydro, CSP, bioenergy, bioenergy with CCS and coal and natural gas with CCS. Oil, natural gas unabated and coal unabated decline to 2050.

The material scope of this study was limited to bulk metals: steel, aluminium and copper, as well as concrete and silicon. The requirements for concrete in energy infrastructure can dwarf those of metals, so though non-metallic, it is important to quantify. Silicon, a metalloid, is not typically categorized in the metals industry but is crucial to PV panels, and its production is highly energy- and CO_2_-intensive. While critical materials such as rare earths, lithium, nickel and silver are indispensable components of the energy transition, their demand has been quantified in literature and in a dedicated report specifically for the IEA NZE scenario [10], alongside recommended actions and overall outlook (bulk materials, however, were not included in this IEA report). The demand for critical and minor materials is at least an order of magnitude lower than the demand for bulk metals steel and aluminium. Wang *et al*. [6] included the full range of minor elements and materials in their analysis and found the CO_2_ contribution of their production to the total to be negligible.

**Figure 3.**
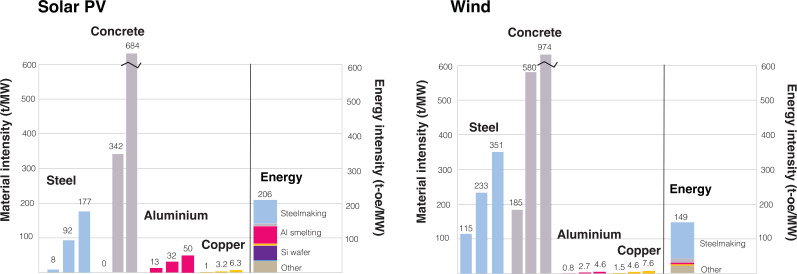
Low-, mid- and high-material intensities (t MW^−1^) for steel, concrete, aluminium and copper in solar PV and wind assumed in the baseline case. The associated energy requirement (in tonne-oil equivalent) per MW of solar PV or wind is also plotted assuming the mid-material intensities.

The quantities of bulk materials required can be estimated using intensities as a function of nameplate capacity (t GW^−1^). The most recent estimates are provided by Kalt *et al*. [7], Wang *et al*. [6] and Deetman *et al*. [3]. We use the mid values from Kalt *et al*. [7] for all electricity generation technologies except solar PV and wind. As solar PV and wind are the dominant technologies, we use a range of lower, mid and upper material intensities provided by Kalt *et al*. [7], with adjustments if intensities provided by Deetman *et al*. [3] fall outside of the range. The mid values by Kalt *et al*. [7] are generally higher relative to Wang *et al*. [6] and Deetman *et al*. [3]: the intensity in Wang *et al*.’s analysis is roughly half of Kalt *et al*. Nevertheless, this range was used to explore the spread in the recent literature. These material intensities are provided in the electronic supplementary data and are plotted for solar PV and wind in figure 3.

Multiplying the material intensities for electricity generation technologies by the capacity projections shown in figure 2 estimates the material stock required for electricity generation. To estimate the quantity of material required to maintain and build up capacity per annum (*inflow*), the capacity that is retiring in that year (*outflow*) must be estimated and replaced, in addition to the planned capacity additions. In the absence of a full vintage stock model, here it is assumed that the PV and wind stock are new in 2021, and the amount of wind and solar PV capacity retiring each year is determined by applying a normal distribution function with an average lifetime of 25 years with a s.d. of 5.35 years [3]. In 2040, the number of wind turbines and solar PV modules coming out of service becomes significant, as shown in figure 2.

The capacity in figure 2 captures electricity generation, but other important components of a net-zero energy system include the electrical grid, storage and low-carbon fuel production and distribution. To capture the associated grid requirements of a net-zero transition, estimates from Kalt *et al*. [7] (mid values from the sustainability + scenario) and Deetman *et al*. [3] (SSSP2 450 scenario) were included. The projections from Kalt *et al*. are significantly higher than Deetman *et al*., so these quantities are used in separate scenarios. The material demands for storage in a net-zero energy system were quantified by Deetman *et al*., who assumed a future portfolio of storage technologies with molten salt batteries, lithium-ion batteries and flow batteries making up the majority. The proportion of steel and aluminium for energy storage was found to be negligible (less than 0.5% of total demand), and thus is not included here. The production capacity of low-carbon fuels (such as biofuels and hydrogen) is projected in the IEA’s net-zero scenario. Due to uncertainty of the infrastructure requirements for hydrogen and biofuel production and distribution, these requirements are not included. It is possible some infrastructure for refining and transporting hydrocarbons may be used. The existing global infrastructure for hydrocarbon extraction, processing and transportation has been characterized by Le Boulzec *et al*. [49], with a model of how this infrastructure may be decommissioned in the IEA’s Net-Zero scenario. The existing hydrocarbon infrastructure is modelled as it is decommissioned and the material becomes available for recycling.

**Figure 4.**
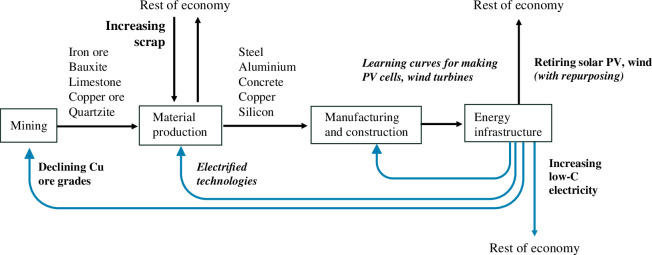
Simple block diagram showing the modelled system. Process steps are shown in the boxes. Black arrows indicate material flows, and the blue arrows indicate energy flows from the energy infrastructure. The trends indicated in bold type are modelled across all scenarios, while those in bold and italicized are explored in dedicated scenarios.

The mining and extraction processes for steel, aluminium, concrete, copper and silicon were modelled, as shown in figure 4. Current mineral and energy requirements of the mining, extraction and refining processes associated with steelmaking, aluminium smelting, copper smelting, concrete production and silicon extraction and refining were established for the year 2021, as informed by the IEA [50] and literature. For example, steelmaking is broken down into BF-BOF, direct reduced iron (DRI) and electric arc furnace (EAF) production routes. The per annum quantity of coke, coal, coke oven gas, BF gas, and electricity used in integrated steelmaking is provided by the IEA [50]. CO_2_ associated with materials production is estimated using CO_2_ intensities of the associated fuel and carbon feedstocks.

Literature estimates were used to model component manufacturing and plant construction. The purification of metallurgical grade silicon to solar grade, followed by ingot casting and slicing to wafers, was informed by Hallam *et al*. [51]. Estimates from Kis *et al*. [52] were used for wind turbine manufacturing and PV plant construction. In all cases, an additional 20% markup (‘other’) was included in the total energy and CO_2_ requirement to account for minor materials and process steps (such as maintenance) that were not included here. The total specific energy requirement (per MW) for building solar PV and wind turbines assuming mid values for material intensity is shown in figure 3.

The materials production system must be modelled dynamically to capture possible changes from now to 2050. Three trends that are underway and well documented are the increasing availability of end-of-life steel, aluminium and copper scrap; declining copper ore grades; and the decreasing average CO_2_ intensity of electricity, as described in the IEA’s net-zero scenario. These changes are modelled in all scenarios. To project the size of the future market for steel, aluminium and concrete the demand projections in the IEA NZE are used. The future demand for copper is estimated using the ‘Policy first’ scenario from Elshkaki *et al*. [53]. The increasing scrap quantities and the shift towards more scrap-based production are estimated using projections from Worldsteel [54], Bertram *et al*. [55] and Elshkaki *et al*. [53]. The global average copper ore grade is projected to decrease from 0.7 to 0.3 wt% from now to 2050, which is assumed to increase the energy intensity of mining and beneficiation by three times [56]. The CO_2_ intensity of electricity decreases according to the global average in the IEA Net-Zero scenario [48] to affect the global average energy intensity and CO_2_ intensity of steel, aluminium, concrete and copper over time.

### (c) Incorporating dynamic improvements

The baseline cases are defined to evaluate if building the infrastructure for the energy transition could be manageable even in the future with no further improvements. Levers to decrease the burden of the transition over time are explored in the scenarios below.

#### (i) Learning curves

The materials intensity of the electricity generation technologies should be modelled dynamically, as the decreasing energy and material requirements to manufacture solar PV modules and wind turbines are well established. The cost of solar PV has fallen by 20% for every doubling in capacity [57], following Wright’s law. Steffen *et al*. [8] and Dale & Benson [9] found that the cost reduction is driven by physical improvements, namely decreasing material use and higher yield per installed capacity, due to longer lifetimes and capacity factors. Energetic experience curves were presented, and an energetic improvement of 19% was found for solar PV and 9% for wind turbines for every doubling of capacity. We incorporate these learning curves into dedicated scenarios. The initial material intensity of solar PV and wind was set to the average between the mid and low values in figure 3, to be better aligned with recent estimates by Deetman *et al*. [3] and Wang *et al*. [6], and reflective of today’s best practices. Wright’s law is then applied to the material intensity values as a function of production capacity with a learning rate of 20% for solar PV and 9% for wind turbines.

#### (ii) Repurposing retiring equipment

The solar PV and wind equipment projected to reach end-of-life before 2050 is appreciable, as shown in figure 2. The rate at which solar PV modules and wind turbines will be decommissioned and repurposed or recycled is highly uncertain, as the current volume of this equipment reaching end-of-life is very low. In the baseline and ‘learning curve’ scenarios, it is assumed that the full volume of decommissioned solar PV and wind turbines must be replaced by wholly new equipment. However, it is possible that these old devices can be repurposed or recycled to reduce the requirement for new materials and manufacturing. In the ‘learning curve and repurposing’ scenario, it is assumed that 70% of the retiring fleet of solar and wind capacity can be repaired or repurposed without any additional material or energy requirements, while the remaining 30% of the fleet is replaced by new.

#### (iii) Electrifying materials production

Each of the material production technologies currently uses electricity to supply some portion of process energy. It is likely that as electricity infrastructure is built and low-carbon electricity becomes more widely available and cheap, these proportions will increase. To model the changing production routes for steelmaking, the increasing volume of scrap arisings is projected to provide 40% of final demand in 2050. It is assumed that the production volume from natural gas DRI grows 1% annually to make up ~10% of the market in 2050. DRI using hydrogen from water electrolysis is assumed to provide 1% of final demand in 2030, 5% in 2040 and 15% in 2050, with linear increases between those milestones. Similarly, MOE is assumed to provide 1% of final demand in 2030, 5% in 2040 and 25% in 2050, with linear increases between those milestones. MSE is assumed to provide 1% of demand in 2040 and 5% in 2050. The BF-BOF route makes up the remaining quantity needed to meet demand (5% of the market in 2050). Aluminium smelting and scrap melting are already electrified. Here, we model the deployment of the inert anode as 1% of the primary market in 2030, 10% in 2040 and 50% in 2050. While there are emerging technologies to fully electrify cement production, the focus of this paper is on metals, so this technology is not explored in detail here. Instead, we assume there is innovation in the electrification of high-temperature heat, with electricity gradually overtaking coal and oil as a thermal power source at clinker kilns. Copper-making is already significantly electrified, with electricity providing ~70% of the process energy in the copper smelting route (80% of copper-making is through pyrometallurgical smelting). We assume that a technology to fully electrify copper production (MSE) is gradually deployed such that 75% of the process energy for smelting is supplied through electricity in 2030, 80% in 2035, 90% in 2040 and 100% in 2045. Hydrometallurgical solvent extraction and electrowinning provide 20% of primary copper demand today, and this proportion is assumed to stay constant. For iron ore, bauxite and limestone mining and beneficiation, the process energy required is assumed to be provided by 80% oil/diesel and 20% electricity today. In this scenario, it is assumed that this gradually shifts to 20% oil/diesel and 80% electricity in 2050.

The key assumptions in the baseline and dynamic improvement scenarios are summarized in table 1.

**Table 1.** Summary of the main assumptions in different scenarios. Across all scenarios, minor materials, low-carbon fuel generation and distribution infrastructure and energy storage were not included in the scope.

assumptions	baseline			dynamic improvements			
				learning curve	learning curve and repurposing		electrification technologies
	low	mid	upper				
material intensity of electricity generation	constant, low (figure 3)	constant, mid (figure 3)	constant, upper (figure 3)	initial value average between low and mid (figure 3). Dynamically updated by Wright’s law for PV and wind			
grid material requirements	Deetman *et al*. [3] SSSP2 450 scenario	Kalt *et al*. [7] mid values in sustainability + scenario		Deetman *et al*. [3] SSSP2 450 scenario			
decommissioning	retiring wind and solar replaced by new equipment				70% of retiring wind and solar can be repurposed without significant new material		
materials production technology	constant, but increasing scrap included, CO_2_ intensity of electricity keeps pace with global average during the transition, and ore grade of copper declines						increased deployment of electrified technologies from 2030 onwards

## 4. Results

The energy and mineral requirements for maintaining the current energy system are summarized in §4a. The material stock and flows to build the energy equipment and infrastructure to achieve the IEA NZE are described in §4b, with the energy and CO_2_ implications described in §4c. The impact of the increased electrification of materials production is summarized in §4d.

### (a) The current energy system

**Figure 5.**
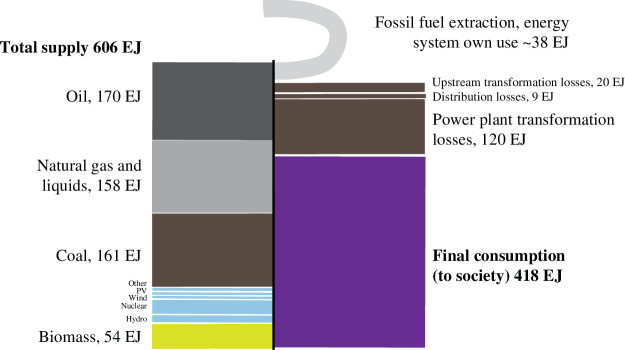
Energy supply and final energy consumption, showing transformation and distribution losses and the energy system’s own use for the year 2019. Data from IEA world energy balance [50].

To establish the current resource flows behind the current energy system, the 2019 global per annum energy inputs and outputs are visualized in figure 5. Fossil fuels, such as coal, oil and natural gas, dominate the energy supply. In transforming this primary energy supply for use outside of the energy industry (final consumption), there is a significant loss, most prominently in the conversion of coal and natural gas to electricity at power plants (120 EJ yr^−1^). The energy system’s own use, as defined by the IEA, includes the energy needed for extraction and distribution and totals about 38 EJ yr^−1^. Indeed, the energy required for oil and gas extraction (10 EJ yr^−1^) and oil refineries (11.6 EJ yr^−1^) is significant, with this breakdown shown in the electronic supplementary data.

Energy is plotted in figure 5, but mass can be attributed as well. Caserini *et al*. [58] report that the per annum flow of fossil fuel minerals is 15 Gt yr^−1^. This roughly equals the global yearly production of all non-energy minerals combined (of which the largest are limestone ~7 Gt yr^−1^, and iron, copper, gold ores each ~2–2.5 Gt yr^−1^).

### (b) Materials for building low-carbon energy infrastructure

**Figure 6.**
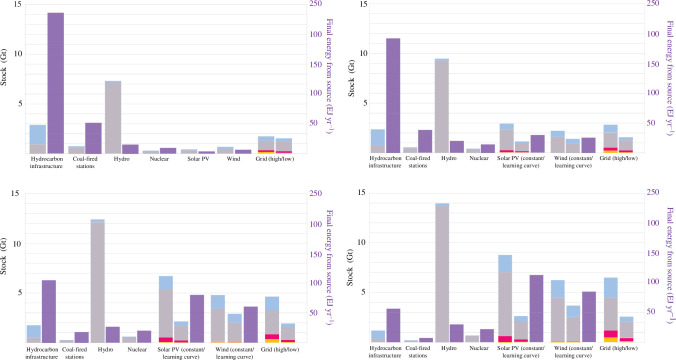
The evolving material stock of energy infrastructure, plotted by type in 2021, 2030, 2040 and 2050. The final energy (final energy consumption as defined by the IEA [48]) delivered from each source is also plotted in purple, corresponding to the right-hand axis. Annual flows, such as fossil fuel minerals, are not plotted. Coal-fired stations include unabated and with CCS.

The evolving material stock of energy infrastructure by type, along with the final energy each source delivers (plotted in purple and corresponding to the right-hand axis), is shown in figure 6. The existing infrastructure for hydrocarbon processing and transportation is gradually decommissioned, potentially supplying ~40–45 Mt yr^−1^ steel by 2050. The large amount of energy delivered from hydrocarbon infrastructure and coal-fired stations compared with their existing material stock may give a misleading impression of material efficiency, as the yearly flux of material is not plotted (~15 Gt yr^−1^ of fossil fuel minerals, as discussed above). Hydroelectricity is the most material-intensive with an immense stock of concrete that is expected to nearly double by 2050. The material stock for the grid, solar PV and wind grow rapidly to 2050, as expected. However, incorporating the learning curve for solar PV and wind turbines reduces the stock significantly. The varying estimates for the build-out of the future grid result in a large range in 2050.

**Figure 7.**
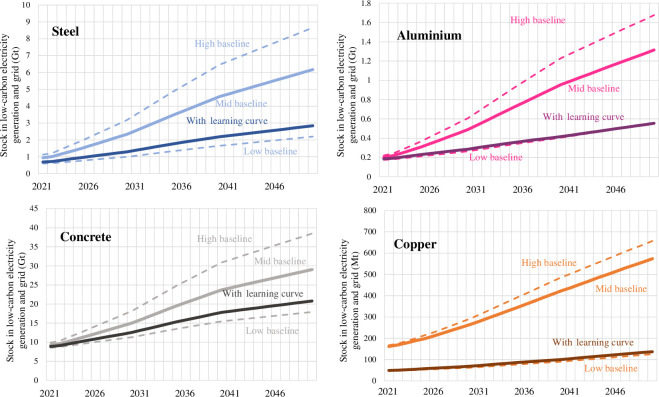
Total steel, aluminium, concrete and copper stock in low-carbon electricity generation equipment (includes hydro, solar PV, wind, bioenergy, bioenergy with CCS, CSP and nuclear) and the grid for the baseline scenarios applying a constant material intensity value (low, mid and upper) and the learning curve scenario.

Figure 7 visualizes the total amounts of steel, aluminium, concrete and copper in low-carbon electricity generation and grid infrastructure for the low-, mid- and upper-intensity values in the constant baseline scenario, as well as when the learning curve is applied. For copper, the assumed grid requirements dominate: the less intensive grid scenario leads to much less copper use in the low and learning curve scenarios. Overall, the range of intensities leads to divergent outcomes in 2050. The wide range of intensities represents the range of designs and practices for building solar PV and wind turbines. Intensity values from recent literature such as Kalt *et al*. [7] are derived from reports and life cycle assessments from different regions over the last 20 years. Updating intensities with the most recent data show greatly improved material use per unit of electricity generated. For example, the total material requirement for utility-scale solar using a 2023 database was found to be 8% of the estimate derived from reports in 2015 [59]. Some disagreement between assessments of the feasibility of the energy transition may be attributed to this range and the difficulty of establishing global average intensities for evolving technologies.

The per annum flows for steel, aluminium, concrete and copper further benchmark the feasibility of building low-carbon electricity infrastructure. For each decade, the maximum yearly flow of steel, aluminium, concrete and copper is plotted for each scenario. In the baseline cases, the material needs increase each decade, driven by capacity build-out, but also to replace retiring wind and solar equipment in 2040–2050. Indeed, the material needs in the mid and upper baseline scenarios are high and would be challenging to support beyond 2030. If a constant mid–high material intensity is applied to 2050 with no recycling or repurposing of retiring infrastructure, steel requirements will be 20–30% of total current steel production, and the requirements for aluminium and copper are close to or exceed the whole of global production today. The proportion of these materials for energy infrastructure compared with projected total demand is shown in the electronic supplementary information but is as high as 20% for steel, 50% for aluminium, 3% for concrete and 45% for copper. However, there is evidence these material intensities may already be outdated [6,59], and a scenario matching the ‘learning curve’ is likely. Repurposing or recycling retiring infrastructure in tandem leads to achievable requirements: ~5% of current per annum steel production, 1–2% of current concrete production, 12–18% of current aluminium production and up to 20% of current copper production. Similarly, the associated mineral requirements, provided in the electronic supplementary information, are high in the 2040–2050 decade mid-baseline case (as high as 340 Mt yr^−1^ iron ore, 210 Mt yr^−1^ limestone, 250 Mt yr^−1^ bauxite and 5.5 Gt yr^−1^ copper ore), but decrease with the learning curve (150 Mt yr^−1^ iron ore, 100 Mt yr^−1^ limestone, 100 Mt yr^−1^ bauxite and 1.5 Gt yr^−1^ copper ore). In all cases, the associated minerals required for this infrastructure are much less than the ~15 Gt yr^−1^ of fossil fuel minerals.

### (c) Energy and CO_2_ implications of building low-carbon energy infrastructure

**Figure 8.**
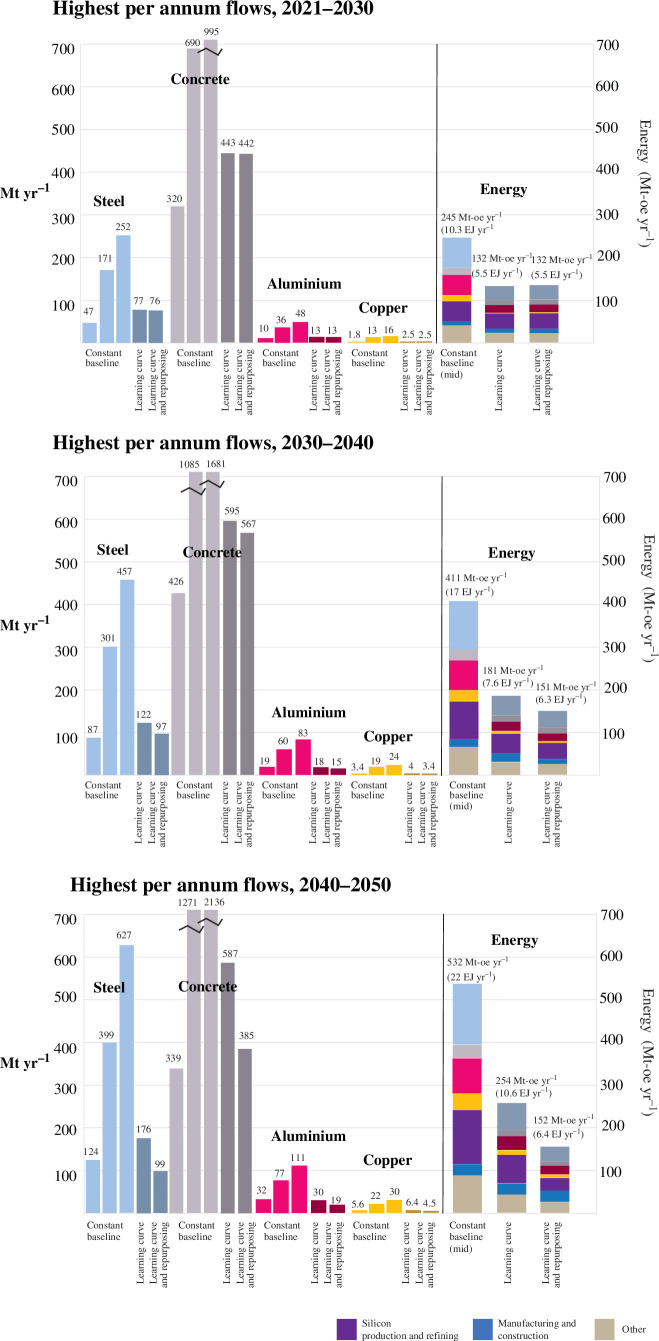
For the decades 2021–2030, 2030–2040 and 2040–2050, the highest per annum flows are plotted for the steel, aluminium, concrete and copper stock required to build low-carbon electricity generation equipment (including hydro, solar PV, wind, bioenergy, bioenergy with CCS, CSP and nuclear) and the grid across the various scenarios (low, mid and upper baseline, learning curve and learning curve with repurposing). The associated energy requirement for producing the materials (steel, concrete, aluminum, copper and silicon), and for construction and manufacturing is plotted to the right for the mid baseline, learning curve and learning curve with repurposing scenarios.

The total energy requirement associated with electricity infrastructure is shown in figure 8 as well, for the baseline (mid), ‘learning curve’ and ‘with repurposing’ cases. The highest value plotted, 22 EJ yr^−1^, is required in the mid-baseline case in 2040–2050. However, this is significantly less than the energy used by the energy industry today (38 EJ yr^−1^; figure 5) to support fossil fuel extraction and processing. In this context, these energy requirements to build lasting infrastructure (*stock*—as shown in figure 6) instead of supporting fossil fuel to be burnt (*flows*—as shown in figure 5) are very attainable, even when considering no future improvements in material production or equipment manufacturing.

Electricity provides ~70% of energy in 2050. The production and distribution of fuels must continue, which is not included in the estimate in figure 8. The IEA NZE quantifies biofuel conversion losses (12–16 EJ yr^−1^) and the energy input/output of hydrogen production (losses of 6–14 EJ yr^−1^) from 2030 to 2050. In addition to the energy required to also support fossil fuel extraction in the near term, care will be needed to balance these energetic losses during the energy transition. It is well documented that the energy required to extract and process fossil fuels is rising [22,60] and carbon capture and storage/utilization can add a further energy requirement of ~30%. The build-out of low-carbon electricity infrastructure should be accompanied by the simultaneous phase-out of fossil fuel extraction not only to limit CO_2_ emissions but also to maintain a reasonable balance of net energy to society [60].

The cumulative CO_2_ emissions (Gt) and energy (EJ) associated with building electricity infrastructure for each scenario are shown in table 2. Kalt *et al*. [7], Deetman *et al*. [3] and Wang *et al*. [6] provide CO_2_ estimates, which are in line with these results. There is a great contrast with Slamersak *et al*. [61], who projected total CO_2_ emissions ranging from 70 to 395 Gt and a 10–34% decline in net energy during the energy transition. Their model incorporated dynamic aspects, such as learning curves and the decreasing CO_2_ intensity of electricity, but was based on EROI ratios with relatively low values for solar PV and wind.

**Table 2.** Sum of CO_2_ (Gt) and energy (EJ) associated with building the stock of low-carbon electricity generation equipment (includes hydro, solar PV, wind, bioenergy, bioenergy with CCS, CSP and nuclear) and the grid to 2050, for each scenario.

	low baseline	mid baseline	high baseline	learning curve	with repurposing	electrified technologies
total CO_2_ (Gt)	9.1	22.8	32.2	11.3	9.6	7.8
total energy requirement (EJ)	232	437	575	206	162	157

Building a low-carbon electricity system will incur CO_2_ emissions, but they constitute a fraction of the remaining carbon budget (total 320 Gt CO_2_-eq for a 1.5°C with 66% avoidance, from the start of 2022 [62]) while providing a real pathway to economy-wide decarbonization. There are levers to minimize associated CO_2_ emissions, but the mechanism is learning by doing. These gains will only emerge upon building and operating and eventually managing and repurposing the infrastructure at end-of-life, not waiting for totally carbon-free manufacturing or energy generation technologies. Overall, the resource requirements and associated CO_2_ emissions should not limit the construction of a net-zero energy system and indeed present an opportunity to greatly reduce total mineral and ‘energy for the energy system’ flows, compared with the current system.

### (d) Impact of electrifying metals production

**Figure 9.**
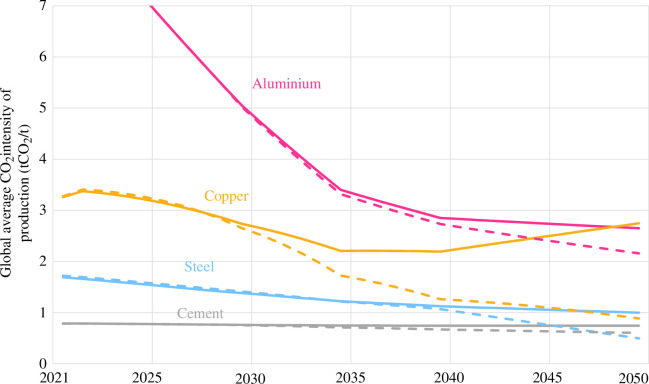
Evolving global average of the CO_2_ intensity of materials production over time in baseline (solid line) and electrified (dashed line) scenarios.

In the baseline scenarios, it is assumed that electricity for material production keeps pace with the global average CO_2_ intensity, resulting in the decreasing CO_2_ intensities for each material shown in figure 9. Aluminium, made from the Hall–Héroult process, exhibits a dramatic decrease in CO_2_ intensity. Supplying aluminium smelters (as well as silicon wafer production, refining and casting, which requires 20 times as much electricity per unit as aluminium) with low-carbon electricity is an urgent priority. Copper mining and smelting are partially electrified, which allows CO_2_ intensity to remain relatively constant until 2050, despite the anticipated tripling of mining and beneficiation energy intensity. Electrifying copper production further allows CO_2_ intensity to decrease while energy consumption increases.

The electrification of processes will help minimize the CO_2_ emissions associated with building new energy infrastructure, as shown in table 2. Applying the electrified technologies across the whole global market yields greater CO_2_ savings, but also reveals the scale of the challenge and the limits of electrification to meeting 2050 carbon goals. The sum of CO_2_ resulting from global production and electricity consumed in each bulk material sector to 2050 is shown in table 3, for the baseline and electrified scenarios.

**Table 3.** Sum of resulting CO_2_ and sum of electricity required for production of each bulk material to 2050, assuming future demand from the IEA NZE and Elshkaki *et al*. [53].

	steel	concrete	aluminium	copper	silicon
CO_2_ from baseline scenario (Gt CO_2_)	74.3	93.2	16.2	2.9	1.8
CO_2_ from electrified scenario (Gt CO_2_)	69.5	87.2	15.3	2.0	
total electricity consumed in baseline scenario (EJ)	158	41	130	32	89
total electricity consumed in electrified scenario (EJ)	243	104	131	46	

Increased electrification of steel production mitigates about 5 Gt CO_2_ to 2050 compared with the baseline but does not slash emissions quickly enough to meet the strict budgets for 1.5°C (38.5 Gt) or 1.7°C (58 Gt) [24]. The electrification scenario presented here is ambitious, projecting the deployment of hydrogen-DRI at 15%, MOE at 25% and MSE at 5% of the steel market in 2050 (for comparison, the IEA NZE projects hydrogen-DRI at 29% and 13% MOE in 2050, with significant carbon capture to close the gap for the carbon budget). The resulting increase in electricity demand for steelmaking is significant: 17 EJ yr^−1^ is required in 2050 compared with ~5 EJ yr^−1^ today, though such an increase is compatible with the IEA NZE. It is likely the global deployment of direct electrification technologies for steelmaking will lag behind the availability of low-carbon electricity. These technologies are nascent, and there is little technical basis for projecting their growth to the scale of hundreds of millions of tonnes. Designing high-throughput electrochemical cells to scale up these concepts will push the frontier of materials science and related fields [34]. Today’s infrastructure build-out should not wait and the total carbon mitigation from their deployment will be marginal in the 2050 timeline, unless a yet more aggressive deployment than the scenario here is anticipated. Nevertheless, unconventional approaches such as MOE and MSE should be nurtured so there is someday a pathway to decouple the manufacture of vial modern infrastructure (energy and otherwise) from CO_2_ emissions using increasingly available low-carbon electricity.

These results confirm the importance of material efficiency and demand reduction, which is the only lever available today to drastically cut emissions associated with material production [63]. Applying low material intensities is a powerful lever to minimize the CO_2_ emissions associated with infrastructure build-out (table 2). Delivering the same service with less material must be a key strategy not only in building energy infrastructure but across all applications [24].

## 5. Discussion: outlook for the coevolution of the metals and energy industries

The metals industry is notoriously risk-averse but must adapt to this changing system. Watari *et al*. [24] advise the heavy industry not to passively wait for infrastructure deployment to begin their decarbonization efforts. However, the metals industry is directly involved in infrastructure build-out. The industry must of course supply key components for energy infrastructure (which needs attention [64]), but they also play an important role as electricity consumers. Aluminium smelters and EAFs are some of the largest single electricity users, with experience that may be shared as other end-users grapple with greater electricity flows. Metals producers can serve as guaranteed buyers to help stabilize the price and supply during the transition. Power purchase agreements are a common mechanism to secure renewable energy at a fixed price in long-term contracts, which steelmakers are increasingly partaking in [31]. Facilities can also commission their own electricity supply. There is great historic precedence for utilizing hydropower for aluminium smelting [65], and harnessing the power from solar farms is underway [66]. If steelmakers pivot towards full electrification, the energy needs will be immense. Birat [25] approximated that a 5 Mt yr^−1^ steel mill will require a 1200 MW power plant or 240 wind turbines. Thus, there may be a strong drive to reduce energy requirements to reduce these capital investments, which may be possible through the development of new chemical routes such as MSE.

The operational challenges of integrating metal production with variable power sources are non-trivial. This integration has been discussed in general terms, but site-by-site dynamic flows must be analysed in detail. High-temperature molten cells will freeze with a power disruption. Innovations in heat and power management such as thermal batteries [67] and thermoelectrics [68] may enable a future where processing industries are ‘swing consumers’, flexibly extracting metal when the sun is shining [69]. Any levers—such as using the electrolytic cell as a battery [70,71], converting sulphur/SO_2_ to electricity—to provide on-demand energy, internally or to the grid, will be crucial. Today, the management of excess heat and hot gases (e.g. gas from the blast furnace and coke oven gas) is optimized for use in downstream operations (e.g. hot rolling) or even separate facilities, such as power plants and for methanol synthesis [72]. This same spirit will be needed to remap supply chains and enable integrated, dynamic use of electricity and heat.

Ramping up the supply of critical metals, including copper, remains a key challenge. The Rocky Mountain Institute views copper ore as a flagship mineral illustrating the opportunities and challenges facing critical materials more broadly [15]. With the demand of copper expected to roughly triple amid declining ore grades—to multiply by up to nine times the already massive flow (~2.5 Gt yr^−1^) of copper ore—a new paradigm is proposed to electrify mining. Implementing renewable energy at remote mine sites (where there is often abundant solar power) requires minimal transmission infrastructure. The Rocky Mountain Institute found the average mine was able to achieve more than 45% electricity decarbonization at a significantly lower cost than the local grid [15]. Electrification efficiency gains at mine sites can help to offset growing energy costs [73]. As touched on in §2, electrification of extraction leads to new chemical conditions, and electrochemical potential can be used to separate species selectively. For example, extracting iron and copper from chalcopyrite in the sulphide regime (MSE), instead of by oxidative smelting, may allow valuable impurities to be extracted upstream. Electro-negative elements such as Ag, Au, Mo, Re and Bi deposit [74] prior to iron and copper, while electro-positive anions such as selenides and tellurides may be decomposed at the anode [43]. The sulphide space hosts promising pathways for extracting critical elements with much greater selectivity [46].

As low-carbon electricity sources, especially intermittent renewables, come online in greater proportion, conventional wisdom about energy usage may require nuanced updating [75]. The minimization of energy consumption is universal common sense, but specific considerations around the time and place of energy use will become paramount. For off-grid, dedicated renewable electricity capacity, there is no operating cost once installed. There may be a new calculus to determine when to do more intensive processing to gain better outcomes for metrics from metal recovery to human health. Could gangue be separated from copper ore using simple phase separation upon melting [76], leaving gangue in a dense, inert form, instead of as tailing dams? Could we spend a bit more energy to extract impurities accumulating in the scrap supply [77]? While raising such possibilities may be naïve when the globe remains decades away from abundant, low-carbon electricity, these cases are arising locally today. Negative wholesale electricity prices are occurring over larger regions with increasing frequency [78] (up to 25% of the time in Kansas and Oklahoma today [79]). Ideally, an energy system is established where using low-carbon electricity is not a negative-sum game, but generation and loads are dynamically matched to best utilize built capacity and lower the costs for all participating.

## 6. Conclusion: navigating near-term constraints, and a long-term vision to make materials with care

Electricity from solar and wind is at cost-parity with electricity from fossil fuels. Building a net-zero energy system could require a considerable fraction of the global metal supply, which may be difficult to maintain beyond 2030 assuming constant mid- or high-material intensities. These apparent limitations are greatly alleviated by factoring in the latest intensity estimates and learning curves. Overall, a low-carbon electricity system should substantially reduce mineral and energy flows compared with maintaining the fossil fuel system, even during the transition. In addition to building new infrastructure, other taxes to the energy system, such as making hydrogen and biofuels, will need to be carefully balanced in the coming decades. While constructing a net-zero energy system is achievable on paper, there are logistical challenges, and the IEA recommends expediting permitting for electricity grids, addressing supply chain bottlenecks and integrating variable renewables [48]. Decommissioning and recycling the vast but often out-of-sight fossil fuel infrastructure will be vital as well [80].

The metals industry has a key reinforcing role to play as a producer and out-sized consumer. The impact of increasingly available low-carbon electricity on material processing will be profound, though difficult to project. Electrification of metal production will be a marginal solution for achieving the 2050 carbon budget. Nevertheless, building the infrastructure will eventually lead to a future of abundant low-carbon electricity: first at specific sites and one day globally. Electrified processes hold promise to meet a host of technical, human health and environmental objectives, including decoupling metals production from CO_2_ emissions.

## Data Availability

All data are available in the electronic supplementary material [81].

## References

[1] Shukla PR (ed). 2022 Climate change 2022: mitigation of climate change. Contribution of Working Group III to the Sixth Assessment Report of the Intergovernmental Panel on Climate Change. Cambridge University Press. (10.1017/9781009157926)

[2] Watari T, McLellan BC, Giurco D, Dominish E, Yamasue E, Nansai K. 2019 Total material requirement for the global energy transition to 2050: a focus on transport and electricity. Resour. Conserv. Recycl. **148**, 91–103. (10.1016/j.resconrec.2019.05.015)

[3] Deetman S, de Boer HS, Van Engelenburg M, van der Voet E, van Vuuren DP. 2021 Projected material requirements for the global electricity infrastructure – generation, transmission and storage. Resour. Conserv. Recycl. **164**, 105200. (10.1016/j.resconrec.2020.105200)

[4] Elshkaki A. 2023 The implications of material and energy efficiencies for the climate change mitigation potential of global energy transition scenarios. Energy **267**, 126596. (10.1016/j.energy.2022.126596)

[5] Månberger A, Stenqvist B. 2018 Global metal flows in the renewable energy transition: exploring the effects of substitutes, technological mix and development. Energy Policy **119**, 226–241. (10.1016/j.enpol.2018.04.056)

[6] Wang S, Hausfather Z, Davis S, Lloyd J, Olson EB, Liebermann L, Núñez-Mujica GD, McBride J. 2023 Future demand for electricity generation materials under different climate mitigation scenarios. Joule **7**, 309–332. (10.1016/j.joule.2023.01.001)

[7] Kalt G, Thunshirn P, Krausmann F, Haberl H. 2022 Material requirements of global electricity sector pathways to 2050 and associated greenhouse gas emissions. J. Clean. Prod. **358**, 132014. (10.1016/j.jclepro.2022.132014)

[8] Steffen B, Hischier D, Schmidt TS. 2018 Historical and projected improvements in net energy performance of power generation technologies. Energy Environ. Sci. **11**, 3524–3530. (10.1039/C8EE01231H)

[9] Dale M, Benson SM. 2013 Energy balance of the global photovoltaic (PV) industry - is the PV industry a net electricity producer? Environ. Sci. Technol. **47**, 3482–3489. (10.1021/es3038824) 23441588

[10] IEA. 2021 The role of critical minerals in clean energy transitions. IEA, Paris. See https://www.iea.org/reports/the-role-of-critical-minerals-in-clean-energy-transitions.

[11] Vidal O, Goffé B, Arndt N. 2013 Metals for a low-carbon society. Nat. Geosci. **6**, 894–896. (10.1038/ngeo1993)

[12] Gailani A, Cooper S, Allen S, Pimm A, Taylor P, Gross R. 2024 Assessing the potential of decarbonization options for industrial sectors. Joule **8**, 576–603. (10.1016/j.joule.2024.01.007)

[13] Khairul MA, Zanganeh J, Moghtaderi B. 2019 The composition, recycling and utilisation of Bayer red mud. Resour. Conserv. Recycl. **141**, 483–498. (10.1016/j.resconrec.2018.11.006)

[14] Saevarsdottir G, Kvande H, Welch BJ. 2020 Reducing the carbon footprint: aluminium smelting with changing energy systems and the risk of carbon leakage (ed. A Tomsett). In Light metals 2020. The Minerals, Metals & Materials Series, pp. 726–734. Cham, Switzerland: Springer. (10.1007/978-3-030-36408-3_98)

[15] Lezak S, Cannon C, Blank TK. 2019 Low-carbon metals for a low-carbon world: a new energy paradigm for mines. Rocky Mountain Institute. See https://rmi.org/wp-content/uploads/2019/12/Low-Carbon_Metals_for_a_Low-Carbon_World.pdf.

[16] Fizaine F, Court V. 2015 Renewable electricity producing technologies and metal depletion: a sensitivity analysis using the EROI. Ecol. Econ. **110**, 106–118. (10.1016/j.ecolecon.2014.12.001)

[17] Harpprecht C, van Oers L, Northey SA, Yang Y, Steubing B. 2021 Environmental impacts of key metals’ supply and low‐carbon technologies are likely to decrease in the future. J. Ind. Ecol. **25**, 1543–1559. (10.1111/jiec.13181)

[18] Krumdieck S. 2020 Transition engineering. Boca Raton, FL: CRC Press/Taylor & Francis.

[19] King LC, van den Bergh JCJM. 2018 Implications of net energy-return-on-investment for a low-carbon energy transition. Nat. Energy **3**, 334–340. (10.1038/s41560-018-0116-1)

[20] Capellán-Pérez I, de Castro C, Miguel González LJ. 2019 Dynamic energy return on energy investment (EROI) and material requirements in scenarios of global transition to renewable energies. Energy. Strat. Rev. **26**, 100399. (10.1016/j.esr.2019.100399)

[21] Vidal O, Rostom F, François C, Giraud G. 2017 Global trends in metal consumption and supply: the raw material–energy nexus. Elements **13**, 319–324. (10.2138/gselements.13.5.319)

[22] Brockway PE, Owen A, Brand-Correa LI, Hardt L. 2019 Estimation of global final-stage energy-return-on-investment for fossil fuels with comparison to renewable energy sources. Nat. Energy **4**, 612–621. (10.1038/s41560-019-0425-z)

[23] Milford RL, Pauliuk S, Allwood JM, Müller DB. 2013 The roles of energy and material efficiency in meeting steel industry CO2 targets. Environ. Sci. Technol. **47**, 3455–3462. (10.1021/es3031424) 23470090

[24] Watari T, Cabrera Serrenho A, Gast L, Cullen J, Allwood J. 2023 Feasible supply of steel and cement within a carbon budget is likely to fall short of expected global demand. Nat. Commun. **14**, 7895. (10.1038/s41467-023-43684-3) 38036547 PMC10689810

[25] Birat JP. 2020 Society, materials, and the environment: the case of steel. Metals **10**, 331. (10.3390/met10030331)

[26] Allanore A. 2012 Contribution of electricity to materials processing: historical and current perspectives. JOM. **65**, 130–135.

[27] Pehl M, Arvesen A, Humpenöder F, Popp A, Hertwich EG, Luderer G. 2017 Understanding future emissions from low-carbon power systems by integration of life-cycle assessment and integrated energy modelling. Nat. Energy **2**, 939–945. (10.1038/s41560-017-0032-9)

[28] Hertwich EG *et al*. 2015 Integrated life-cycle assessment of electricity-supply scenarios confirms global environmental benefit of low-carbon technologies. Proc. Natl Acad. Sci. USA **112**, 6277–6282. (10.1073/pnas.1312753111) 25288741 PMC4443343

[29] Bhaskar A, Assadi M, Nikpey Somehsaraei H. 2020 Decarbonization of the iron and steel industry with direct reduction of iron ore with green hydrogen. Energies **13**, 758. (10.3390/en13030758)

[30] Perpiñán J, Peña B, Bailera M, Eveloy V, Kannan P, Raj A, Lisbona P, Romeo LM. 2023 Integration of carbon capture technologies in blast furnace based steel making: a comprehensive and systematic review. Fuel **336**, 127074. (10.1016/j.fuel.2022.127074)

[31] Birat JP. 2023 Net-zero transition in the steel sector: beyond the simple emphasis on hydrogen, did we miss anything? Mat. Tech. **111**, 201. (10.1051/mattech/2023003)

[32] Kim W, Sohn I. 2022 Critical challenges facing low carbon steelmaking technology using hydrogen direct reduced iron. Joule **6**, 2228–2232. (10.1016/j.joule.2022.08.010)

[33] Allanore A. 2015 Features and challenges of molten oxide electrolytes for metal extraction. J. Electrochem. Soc. **162**, E13–E22. (10.1149/2.0451501jes)

[34] Allanore A. 2017 Electrochemical engineering for commodity metals extraction. Electrochem. Soc. Interface **26**, 63–68. (10.1149/2.F05172if)

[35] Yuan B, Kongstein OE, Haarberg GM. 2008 Electrowinning of iron in aqueous alkaline solution using a rotating cathode. J. Electrochem. Soc. **156**, D64. (10.1149/1.3039998)

[36] Olsen K, Van der Laan S, Lavelaine de Maubeuge H, Serna M *et al*. 2016 Iron production by electrochemical reduction of its oxide for high CO_2_ mitigation (IERO). European Commission: Directorate-General for Research and Innovation. See https://data.europa.eu/doi/10.2777/084034.

[37] Sadoway DR. 1991 The electrochemical processing of refractory metals. JOM **43**, 15–19. (10.1007/BF03220614)

[38] Wang D, Gmitter AJ, Sadoway DR. 2011 Production of oxygen gas and liquid metal by electrochemical decomposition of molten iron oxide. J. Electrochem. Soc. **158**, E51. (10.1149/1.3560477)

[39] Allanore A, Yin L, Sadoway DR. 2013 A new anode material for oxygen evolution in molten oxide electrolysis. Nature **497**, 353–356. (10.1038/nature12134) 23657254

[40] Taylor PR, Wang W. 2002 A laboratory investigation of the reduction of chromium oxide by a reverse-polarity DC plasma-driven molten oxide electrolysis process. Plasma Chem. Plasma Process. **22**, 387–400. (10.1023/A:1015317116090)

[41] Ferreira NM, Kovalevsky AV, Ferro MC, Costa FM, Frade JR. 2016 A new concept of ceramic consumable anode for iron pyroelectrolysis in magnesium aluminosilicate melts. Ceram. Int. **42**, 11070–11076. (10.1016/j.ceramint.2016.04.004)

[42] Sokhanvaran S, Lee SK, Lambotte G, Allanore A. 2016 Electrochemistry of molten sulfides: copper extraction from BaS-Cu_2_. J. Electrochem. Soc. **163**, D115–D120. (10.1149/2.0821603jes)

[43] Daehn KE, Stinn C, Rush L, Benderly-Kremen E, Wagner ME, Boury C, Chmielowiec B, Gutierrez C, Allanore A. 2022 Liquid copper and iron production from chalcopyrite, in the absence of oxygen. Metals **12**, 1440. (10.3390/met12091440)

[44] Stinn C, Gutierrez C, Daehn KE, Allanore A. 2022 Sulfidation for copper mineral processing and impurity management. In Copper International Conference, Santiago, Chile.

[45] Suryarao KP. 2024 Iron production by molten sulfide electrolysis. Master's Thesis, Massachusetts Institute of Technology. https://dspace.mit.edu/handle/1721.1/155400.

[46] Stinn C, Allanore A. 2022 Selective sulfidation of metal compounds. Nature **602**, 78–83. (10.1038/s41586-021-04321-5) 34915548

[47] International Energy Agency (IEA). 2019 Extended world energy balance. See https://www.iea.org/data-and-statistics/data-product/world-energy-balances.

[48] International Energy Agency (IEA). 2023 Net zero roadmap: A global pathway to keep the 1.5. See https://www.iea.org/reports/net-zero-roadmap-a-global-pathway-to-keep-the-15-0c-goal-in-reach.

[49] Le Boulzec H, Delannoy L, Andrieu B, Verzier F, Vidal O, Mathy S. 2022 Dynamic modeling of global fossil fuel infrastructure and materials needs: overcoming a lack of available data. Appl. Energy **326**, 119871. (10.1016/j.apenergy.2022.119871)

[50] International Energy Agency. 2022 World energy statistics and balances. See https://www.iea.org/data-and-statistics/data-product/world-energy-statistics-and-balances.

[51] Hallam B, Kim M, Underwood R, Drury S, Wang L, Dias P. 2022 A polysilicon learning curve and the material requirements for broad electrification with photovoltaics by 2050. Solar RRL **6**, 1–8. (10.1002/solr.202200458)

[52] Kis Z, Pandya N, Koppelaar RHEM. 2018 Electricity generation technologies: comparison of materials use, energy return on investment, jobs creation and CO_2_ emissions reduction. Energy Policy **120**, 144–157. (10.1016/j.enpol.2018.05.033)

[53] Elshkaki A, Graedel TE, Ciacci L, Reck BK. 2016 Copper demand, supply, and associated energy use to 2050. Glob. Environ. Change **39**, 305–315. (10.1016/j.gloenvcha.2016.06.006)

[54] Worldsteel Association. 2021 Scrap use in the steel industry. See https://worldsteel.org/wp-content/uploads/Fact-sheet-on-scrap_2021.pdf.

[55] Bertram M, Ramkumar S, Rechberger H, Rombach G, Bayliss C, Martchek KJ, Müller DB, Liu G. 2017 A regionally-linked, dynamic material flow modelling tool for rolled, extruded and cast aluminium products. Resour. Conserv. Recycl. **125**, 48–69. (10.1016/j.resconrec.2017.05.014)

[56] Calvo G, Mudd G, Valero A, Valero A. 2016 Decreasing ore grades in global metallic mining: a theoretical issue or a global reality? Res. **5**, 36. (10.3390/resources5040036)

[57] Roser M. 2023 Learning curves: What does it mean for a technology to follow Wright’s law?. See https://ourworldindata.org/cheap-renewables-growth.

[58] Caserini S, Storni N, Grosso M. 2022 The availability of limestone and other raw materials for ocean alkalinity enhancement. Global Biogeochem. Cycles **36**. (10.1029/2021GB007246)

[59] Wang S, Cook P, Stein A, Lloyd J, Smith C. 2024 Updated mining footprints and raw material needs for clean energy. See https://thebreakthrough.org/issues/energy/updated-mining-footprints-and-raw-material-needs-for-clean-energy.

[60] Delannoy L, Longaretti PY, Murphy DJ, Prados E. 2021 Peak oil and the low-carbon energy transition: a net-energy perspective. Appl. Energy **304**, 117843. (10.1016/j.apenergy.2021.117843)

[61] Slameršak A, Kallis G, O’Neill DW. 2022 Energy requirements and carbon emissions for a low-carbon energy transition. Nat. Commun. **13**, 6932. (10.1038/s41467-022-33976-5) 36376312 PMC9663537

[62] Friedlingstein P *et al*. 2022 Global carbon budget 2021. Earth Syst. Sci. Data **14**, 1917–2005. (10.5194/essd-14-1917-2022)

[63] Allwood JM, Cullen JM, Milford RL. 2010 Options for achieving a 50% cut in industrial carbon emissions by 2050. Environ. Sci. Technol. **44**, 1888–1894. (10.1021/es902909k) 20121181

[64] U.S. Department of Energy, Office of Policy. 2022 The supply chain crisis facing the nation’s electric grid. See https://www.energy.gov/policy/articles/supply-chain-crisis-facing-nations-electric-grid.

[65] Ali S. 2023 Soil to foil: aluminum and the quest for industrial sustainability. New York, NY; Chichester, UK: Columbia University Press.

[66] Hoyle R. 2024 Wall street journal. Rio tinto inks power deal with european energy for giant australia solar farm. See https://www.wsj.com/finance/rio-tinto-inks-power-deal-with-european-energy-for-giant-australia-solar-farm-aee1395d.

[67] Gur I, Sawyer K, Prasher R. 2012 Engineering. searching for a better thermal battery. Science **335**, 1454–1455. (10.1126/science.1218761) 22442472

[68] Zhao Y, Rinzler C, Allanore A. 2017 Molten semiconductors for high temperature thermoelectricity. ECS J. Solid State Sci. Technol. **6**, 3010–N3016. (10.1149/2.0031703jss)

[69] Lechtenböhmer S, Nilsson LJ, Åhman M, Schneider C. 2016 Decarbonising the energy intensive basic materials industry through electrification – Implications for future EU electricity demand. Energy **115**, 1623–1631. (10.1016/j.energy.2016.07.110)

[70] Deen KM, Asselin E. 2020 Integration of Cu extraction and Zn electrowinning processes for energy storage. J. Clean. Prod. **253**, 119779. (10.1016/j.jclepro.2019.119779)

[71] Deen KM, Asselin E. 2019 On the use of a naturally-sourced cufes2 mineral concentrate for energy storage. Electrochim. Acta **297**, 1079–1093. (10.1016/j.electacta.2018.11.178)

[72] Razzaq R, Li C, Zhang S. 2013 Coke oven gas: availability, properties, purification, and utilization in China. Fuel **113**, 287–299. (10.1016/j.fuel.2013.05.070)

[73] Igogo T, Awuah-Offei K, Newman A, Lowder T, Engel-Cox J. 2021 Integrating renewable energy into mining operations: opportunities, challenges, and enabling approaches. Appl. Energy **300**, 117375. (10.1016/j.apenergy.2021.117375)

[74] Sahu SK, Chmielowiec B, Allanore A, Member ISE. 2017 Electrolytic extraction of copper, molybdenum and rhenium from molten sulfide electrolyte. Electrochim. Acta **243**, 382–389. (10.1016/j.electacta.2017.04.071)

[75] Diesendorf M, Wiedmann T. 2020 Implications of trends in energy return on energy invested (EROI) for transitioning to renewable electricity. Ecol. Econ. **176**, 106726. (10.1016/j.ecolecon.2020.106726)

[76] Daehn K, Benderly-Kremen E, Yagi R, Stinn C, Boury C, Rush L, Wagner ME, Allanore A. 2022 Scaling up molten sulfide electrolysis for liquid copper production from chalcopyrite. In Copper International Conference, Santiago, Chile.

[77] Daehn KE, Serrenho AC, Allwood J. 2019 Finding the most efficient way to remove residual copper from steel scrap. Metall. Mater. Trans. B **50**, 1225–1240. (10.1007/s11663-019-01537-9)

[78] Bajwa BM, Cavicchi J. 2017 Growing evidence of increased frequency of negative electricity prices in U.S. wholesale electricity markets. IAEE Energy Forum (Fourth Quarter) 37–41.

[79] Seel J, Millstein D, Mills A, Bolinger M, Wiser R. 2021 Plentiful electricity turns wholesale prices negative. Adv. Appl. Energy **4**, 100073. (10.1016/j.adapen.2021.100073)

[80] Partridge T, Barandiaran J, Triozzi N, Toni Valtierra V. 2023 Decommissioning: another critical challenge for energy transitions. Glob. Soc. Chall. J. **2**, 188–202. (10.1332/NNBM7966)

[81] Daehn K, Allanore A, Olivetti E. 2024 Data from: A key feedback loop: building electricity infrastructure and electrifying metals production. Figshare. (10.6084/m9.figshare.c.7484138)39489175

